# Comparison of Machine Learning Methods Using Spectralis OCT for Diagnosis and Disability Progression Prognosis in Multiple Sclerosis

**DOI:** 10.1007/s10439-022-02930-3

**Published:** 2022-02-26

**Authors:** Alberto Montolío, José Cegoñino, Elena Garcia-Martin, Amaya Pérez del Palomar

**Affiliations:** 1 Group of Biomaterials, Aragon Institute of Engineering Research (I3A), University of Zaragoza, Zaragoza, Spain; 2 Department of Mechanical Engineering, University of Zaragoza, Zaragoza, Spain; 3 Ophthalmology Department, Miguel Servet University Hospital, Zaragoza, Spain; 4 GIMSO Research and Innovative Group, Aragon Institute for Health Research (IIS Aragon), Zaragoza, Spain; 5 Escuela de Ingeniería y Arquitectura, Campus Río Ebro, Edificio Betancourt, C/Maria de Luna s/n, 50018 Zaragoza, Spain

**Keywords:** Multiple sclerosis, Machine learning, Optical coherence tomography, Retinal nerve fiber layer

## Abstract

Machine learning approaches in diagnosis and prognosis of multiple sclerosis (MS) were analysed using retinal nerve fiber layer (RNFL) thickness, measured by optical coherence tomography (OCT). A cross-sectional study (72 MS patients and 30 healthy controls) was used for diagnosis. These 72 MS patients were involved in a 10-year longitudinal follow-up study for prognostic purposes. Structural measurements of RNFL thickness were performed using different Spectralis OCT protocols: fast macular thickness protocol to measure macular RNFL, and fast RNFL thickness protocol and fast RNFL-N thickness protocol to measure peripapillary RNFL. Binary classifiers such as multiple linear regression (MLR), support vector machines (SVM), decision tree (DT), k-nearest neighbours (k-NN), Naïve Bayes (NB), ensemble classifier (EC) and long short-term memory (LSTM) recurrent neural network were tested. For MS diagnosis, the best acquisition protocol was fast macular thickness protocol using k-NN (accuracy: 95.8%; sensitivity: 94.4%; specificity: 97.2%; precision: 97.1%; AUC: 0.958). For MS prognosis, our model with a 3-year follow up to predict disability progression 8 years later was the best predictive model. DT performed best for fast macular thickness protocol (accuracy: 91.3%; sensitivity: 90.0%; specificity: 92.5%; precision: 92.3%; AUC: 0.913) and SVM for fast RNFL-N thickness protocol (accuracy: 91.3%; sensitivity: 87.5%; specificity: 95.0%; precision: 94.6%; AUC: 0.913). This work concludes that measurements of RNFL thickness obtained with Spectralis OCT have a good ability to diagnose MS and to predict disability progression in MS patients. This machine learning approach would help clinicians to have valuable information.

## Introduction

Multiple sclerosis (MS) is a chronic inflammatory demyelinating autoimmune disease of the central nervous system (CNS) in which axonal loss is considered the main cause of disability.^61^ Despite its high heterogeneity and unpredictable course, this disease is characterized by relapses with reversible neurological problems. After each relapse, a gradual neurological worsening is often observed.^34^

Axonal damage in MS patients is also widespread in the neuroretina. The visual pathway is one of the most affected systems, where inflammation, demyelination and axonal degeneration cause visual symptoms. This fact highlights the importance of studying neuroretina as a possible MS biomarker.^13,45^ Optical coherence tomography (OCT) is a non-invasive, objective and reproducible method to monitor retinal damage. OCT devices provide measurements of each retinal layer and, therefore, show great potential for quantifying axonal damage by measuring peripapillary retinal nerve fiber layer (pRNFL) and macular RNFL (mRNFL) thicknesses.^30^

The use of Fourier-domain OCT (FD-OCT) provided higher resolution in relation to time-domain OCT (TD-OCT), which required long acquisition times. FD-OCT is divided into spectral-domain OCT (SD-OCT) and swept-source OCT (SS-OCT). Current SD-OCT and SS-OCT devices use lasers of different wavelengths to acquire OCT images in the same way. Some commercially available SD-OCT devices are RTVue (Optovue, Fremont, CA, USA), Spectralis OCT (Heidelberg Engineering, Heidelberg, Germany), SOCT Copernicus (Optopol Technology, Zawiercie, Poland), Cirrus HD-OCT (Carl Zeiss Meditec, Dublin, CA, USA), and 3D OCT-1000 (Topcon, Paramus, NJ, USA). And others with SS-OCT technology are Triton SS-OCT (Topcon, Tokyo, Japan) and Plex Elite 9000 (Carl Zeiss Meditec, Dublin, CA, USA).

OCT technique allows correlating retinal neurodegeneration and MS disability.^2,12, 55^ In this way, some authors demonstrated its potential, in combination with artificial intelligence (AI), as an early diagnostic tool. Garcia-Martin *et al*.^16^ applied artificial neural network (ANN) to pRNFL thickness in order to analyse the ability of Spectralis OCT to diagnose MS. Cavaliere *et al*.^9^ designed a computer-aided diagnosis method using support vector machine (SVM) with mRNFL and pRNFL measurements performed by Triton SS-OCT from 48 MS patients and 48 healthy controls. These database was also used by Garcia-Martin *et al*.^17^ with a feed-forward neural network as a deep learning technique. Pérez del Palomar *et al*.^41^ used machine learning techniques for MS diagnosis using mRNFL and pRNFL thicknesses measured by Triton SS-OCT. With a sample of 80 MS patients and 180 healthy controls, the results were promising with an accuracy of 97.2% using decision tree (DT) and mRNFL.

To analyse disability progression, there are two approaches. The first approach is to observe whether secondary-progressive (SPMS) development occurs in patients with relapse-remitting type (RRMS) such that the neurological state continues to worsen. The second approach, which is widely used, is based on the variation of expanded disability status scale (EDSS). This scale ranges from 0 (healthy control) to 10 (patient died from MS).^31,56^ The disability progression depends on the EDSS measurement as a reference and the EDSS variation (∆EDSS) over time, these standard criteria represent a relevant worsening of disability state.^22^

Most studies have based their prognosis on correlations and statistical analysis. Rothman *et al*.^47^ showed that lower baseline macular volume was associated with higher 10-year EDSS scores. Paying attention to RNFL thickness, the study conducted by Schurz *et al*.^51^ demonstrated how a pRNFL thinning > 1.5 µm/year was related to a higher likelihood of disability worsening. The same annual pRNFL thinning rate was used to discriminate between stable and progressing MS patients, and was associated with a 15-fold increased risk of disability progression.^8^ Moreover, a baseline pRNFL thickness <88 µm was reflected in a 3-fold increased risk of EDSS progression.^7^ Also ganglion cell-inner plexiform layer (GCIPL) showed promise for this purpose, where a baseline GCIPL thickness < 70 µm was independently associated with long-term disability worsening in MS.^26^ Similar result was obtained by Bsteh *et al*.^6^ who set the baseline macular GCIPL (mGCIPL) barrier at 77 µm and the annual mGCIPL loss rate barrier at 1 µm. Other authors reached the same conclusion, showing that GCIPL thinning >1 µm/year represented an increased risk of disability worsening.^51^

As shown above, several longitudinal studies demonstrated the relationship between RNFL thickness and disability progression. After proving the good performance of AI with OCT data for the diagnosis of this disease, the next step could be to apply AI using OCT data for MS prognosis. However, more recent studies have limited the data to those obtained using the tests included in McDonald criteria such as magnetic resonance imaging (MRI) or evoked potential (EP)^53^. Zhao *et al*.^65^ compared SVM, logistic regression (LR), random forest (RF) and several ensemble classifiers (EC) to predict ∆EDSS up to 5 years after the baseline using MRI data acquired in the first 2 years. The work performed by Yperman *et al*.^62^ used RF and LR to predict disability progression after 2 years using 2-year EP time series. Seccia *et al*.^52^ predicted whether the disease would progress from RRMS to SPMS applying different machine learning approaches to MRI and Liquor analysis data from the last available visit or the whole clinical history. Another study evaluated LR, SVM, DT and EC for MS prognosis between 2-year follow-up and baseline also using MRI data.^27^

As can be seen, previous studies predicted disability progression in the short term and did not focus machine learning approaches on OCT data for MS prognosis.^40^ However, we proposed the use of AI to predict long-term disability state using OCT data. In our previous work,^36^ RNFL thickness measured by Cirrus HD-OCT showed a high performance for MS prognosis. Along the same lines, in this work, different AI approaches were applied to RNFL thicknesses measured by Spectralis OCT in order to analyse which acquisition protocol and which classifier works best for predicting disability progression in the long term.

## Material and Methods

### Study Population

The study procedure was approved by the Ethics Committee of Clinic Research in Aragon (CEICA) and by the Ethics Committee of Miguel Servet University Hospital (Zaragoza, Spain). This work was performed in accordance with the tenets of the Declaration of Helsinki. All participants provided written informed consent to participate in the study.

This work includes a cross-sectional study and a longitudinal study. The cross-sectional study enrolled 72 MS patients (19 males and 53 females) and 30 healthy controls (5 males and 25 females). The age of MS patients ranged from 25 to 72 years with a mean of 45.94 years, while for healthy controls if ranged from 26 to 73 with a mean of 48.78 years (see Table 1). MS patients were diagnosed by a neurologist based on the 2010 revision of the McDonald Criteria ^43^. In the longitudinal study, 72 MS patients were evaluated at several visits until the 10-year follow-up was completed. The participants had no concomitant ocular diseases, nor any history of retinal pathology or systemic conditions that could affect the visual function. All participants underwent neuro-ophthalmological evaluations, including best-corrected visual acuity (BCVA) to quantify the level of vision and EDSS to register MS-associated disability.

**Table 1:** General data and retinal nerve fiber layer (RNFL) data, measured by Spectralis optical coherence tomography (OCT), from 72 patients with multiple sclerosis (MS) and 30 healthy controls.

	MS patients (*n *= 72)	Healthy controls (*n *= 30)	*p*-value
General data			
Age [years]	45.94 ± 10.85	48.78 ± 14.70	0.247
Sex [M–F]	19–53	5–25	
BCVA [Snellen]	0.90 ± 0.24	1.00 ± 0.11	0.020
Fast macular thickness protocol			
Total volume [mm^3^]	0.78 ± 0.16	0.96 ± 0.11	< 0.001
Central fovea th. [*μ*m]	12.32 ± 2.78	12.40 ± 1.77	0.818
Inner nasal th. [*μ*m]	19.83 ± 4.86	22.50 ± 2.70	< 0.001
Outer nasal th. [*μ*m]	39.89 ± 13.68	52.53 ± 6.82	< 0.001
Inner superior th. [*μ*m]	21.32 ± 4.18	24.43 ± 2.11	< 0.001
Outer superior th. [*μ*m]	31.15 ± 7.36	38.50 ± 5.10	< 0.001
Inner temporal th. [*μ*m]	17.65 ± 2.06	17.97 ± 1.52	0.488
Outer temporal th. [*μ*m]	18.31 ± 2.14	19.67 ± 1.94	< 0.001
Inner inferior th. [*μ*m]	22.50 ± 4.46	27.13 ± 3.20	< 0.001
Outer inferior th. [*μ*m]	31.69 ± 8.13	40.70 ± 7.95	< 0.001
Fast RNFL thickness protocol			
Mean th. [*μ*m]	84.39 ± 15.15	101.37 ± 8.60	< **0.001**
Temporal th. [*μμμμ*m]	59.53 ± 16.69	73.13 ± 12.85	< **0.001**
Superotemporal th. [*μ*m]	115.36 ± 23.50	134.77 ± 19.87	**0.001**
Inferotemporal th. [*μ*m]	118.78 ± 27.44	146.40 ± 19.00	< **0.001**
Nasal th. [*μ*m]	67.56 ± 18.39	75.63 ± 12.73	**0.005**
Superonasal th. [*μ*m]	90.38 ± 21.85	118.20 ± 27.33	< **0.001**
Inferonasal th. [*μ*m]	96.51 ± 26.95	115.00 ± 17.88	< **0.001**
Fast RNFL-N thickness protocol			
Mean th. [*μ*m]	83.77 ± 17.69	101.55 ± 9.69	0.172
PMB th. [*μ*m]	44.73 ± 12.91	55.83 ± 8.84	< **0.001**
N/T ratio	1.29 ± 0.46	1.14 ± 0.35	0.082
Superonasal th. [*μ*m]	96.62 ± 27.13	107.34 ± 22.90	**0.010**
Nasal th. [*μ*m]	67.61 ± 19.73	79.62 ± 15.36	< **0.001**
Inferonasal th. [*μ*m]	100.68 ± 28.01	118.14 ± 24.77	**0.005**
Inferotemporal th. [*μ*m]	114.25 ± 29.19	147.72 ± 23.17	< **0.001**
Temporal th. [*μ*m]	55.15 ± 14.87	73.03 ± 13.35	< **0.001**
Superotemporal th. [*μ*m]	112.79 ± 28.63	135.28 ± 18.39	< **0.001**

*p *value, based on Wilcoxon test, is used to compare data between MS patients and healthy controls. Statistically significant differences (*p *< 0.05) are represented in bold

*BCVA* best-corrected visual acuity, *th* thickness, *PMB* papillomacular bundle, *N/T* nasal/temporal

The required inclusion criteria were: BCVA of 20/40 or higher, refractive error within ±5.00 diopters equivalent sphere and ± 2.00 diopters astigmatism, transparent ocular media (nuclear colour/opalescence, cortical or posterior subcapsular lens opacity <1), according to the Lens Opacities Classification System III^11^. From these 102 subjects of white European origin, one randomly selected eye was analysed to avoid potential bias by interrelation between eyes of the same subject. In subjects with exclusion criteria in one eye, the other eye was selected.

### OCT Evaluation

Structural measurements of RNFL were performed using the Spectralis OCT (Heidelberg Engineering, Inc., Heidelberg, Germany). The Spectralis OCT uses a blue quality bar in the image to indicate signal strength. The quality score ranges from 0 (poor quality) to 40 (excellent quality). Only images with quality higher than 25 were analysed. A real-time eye-tracking system measures eye movements and provides feedback to the scanning mechanism to stabilize the retinal position on the B-scan. This system enables sweep averaging at each B-scan location to reduce speckle noise. The average number of scans to produce each circular B-scan was nine. The TruTrack eye tracking technology (Heidelberg Engineering) recognizes, locks onto, follows the patient’s retina during scanning and automatically places follow-up scans to ensure accurate monitoring of disease progression.^19^ All scans were obtained by operators with extensive experience in the use of the OCT device. Databases were performed in accordance to the quality control criteria (OSCAR-IB) and the Advised Protocol for OCT Study Terminology and Elements (APOSTEL) criteria.^14,50^

**Figure 1:**
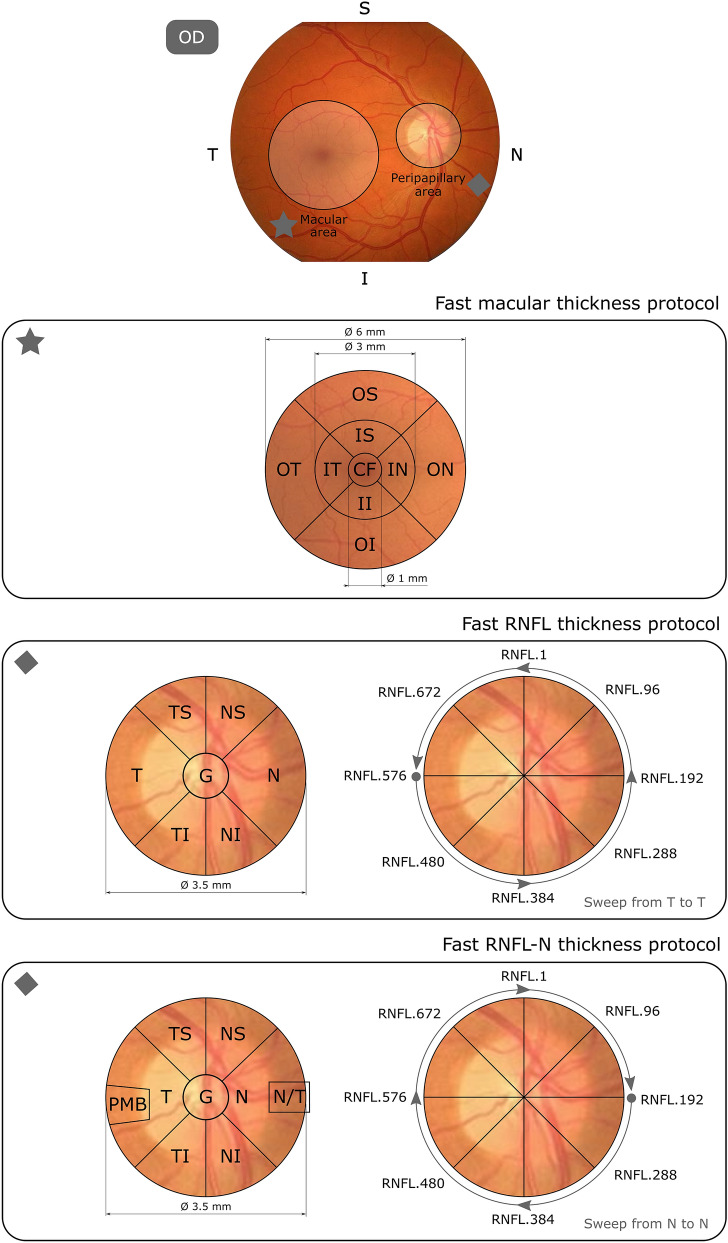
Schematic representation of Spectralis OCT acquisition protocols on a right eye retina. Fast macular thickness protocol measures total volume and macular RNFL (mRNFL) thickness in nine sectors (*CF* central fovea, *IN* inner nasal, *ON* outer nasal, *IS* inner superior, *OS* outer superior, *IT* inner temporal, *OT* outer temporal, *II* inner inferior, *OI* outer inferior). Fast RNFL thickness protocol measures peripapillary RNFL (pRNFL) thickness by providing mean pRNFL thickness (G) and pRNFL thickness in six sectors *NS* superonasal, *N* nasal, *NI* inferonasal, *TI* inferotemporal, *T* temporal, *TS* superotemporal. This protocol also generates 768 pRNFL thickness measurements with a circular sweep from temporal to temporal (counterclockwise). Fast RNFL-N thickness protocol differs from the previous one in two aspects: it adds papillomacular bundle (PMB) thickness and nasal/temporal (N/T) ratio, and the circular sweep is performed from nasal to nasal (clockwise). *OD* right eye, *OCT* optical coherence tomography, *RNFL* retinal nerve fiber layer.

This OCT device allows to measure the RNFL thickness in different areas depending on the protocol used (see Fig. 1). Spectralis OCT directly provides RNFL thickness data from OCT images. This automated segmentation was performed with the manufacturer’s software Heidelberg Eye Explorer (HEYEX) which consist of a multilayer segmentation algorithm (Heidelberg Engineering). **Fast macular thickness protocol**: a map around the fovea showing the total volume and the mRNFL thickness in nine sectors (central fovea, inner nasal, outer nasal, inner superior, outer superior, inner temporal, outer temporal, inner inferior and outer inferior). Three concentric circles (1 mm, 3 mm and 6 mm) define these nine macular sectors established by the Early Treatment Diabetic Retinopathy Study (ETDRS). **Fast RNFL thickness protocol**: a 3.5 mm diameter circle scan centred on the optic disc showing the mean pRNFL thickness and the pRNFL thickness in six sectors (superonasal, nasal, inferonasal, inferotemporal, temporal and superotemporal). This protocol also generates a database with pRNFL thickness measurements at all 768 points registered during circular peripapillary scan acquisition. The image sweep is done from temporal to temporal. **Fast RNFL-N thickness protocol:** like fast RNFL thickness protocol, a map around the optic disc with the mean pRNFL thickness and the pRNFL thickness in six sectors, and also the 768 sector pRNFL thicknesses. Two extra data are the papillomacular bundle (PMB) thickness and nasal/temporal (N/T) ratio. In this protocol, unlike fast RNFL thickness protocol, the sweep is done from nasal to nasal.

### Statistical Analysis

Statistical analysis was performed with Matlab (version 2020b, Mathworks Inc., Natick, MA). The Kolmogorov-Smirnov test was used to analyse the normality of numerical variables. Comparison between groups was performed using the Wilcoxon test as an alternative to the Student’s *t*-test due to the non-normality of the variables. A *p*-value < 0.05 was considered statistically significant.

### Machine Learning Pipeline

**Figure 2:**
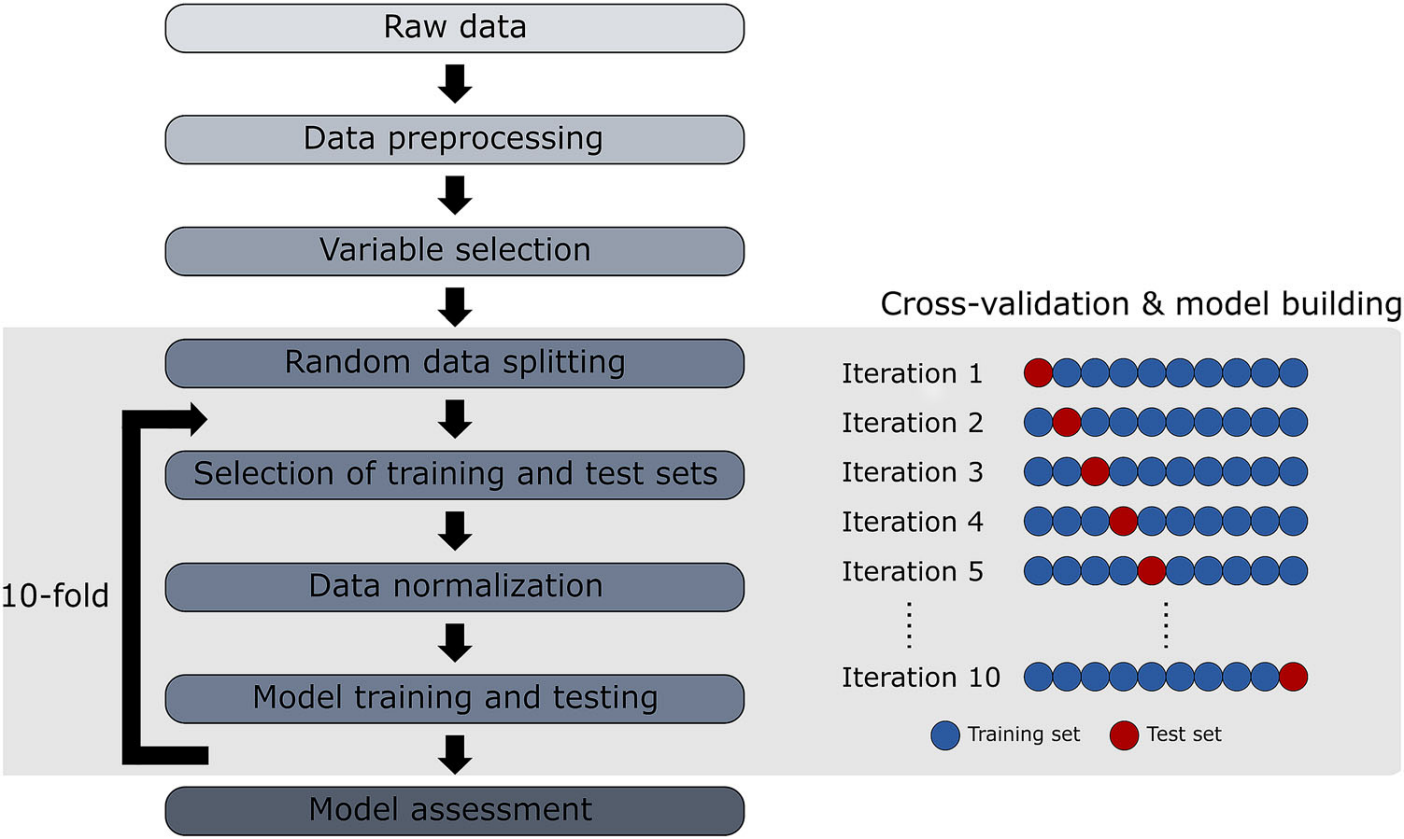
Machine learning pipeline of the proposed method consists of five steps: data preprocessing, variable selection, 10-fold cross-validation, model building and model assessment.

The aim of this work was to diagnose MS disease and predict the disability progression in MS patients using clinical data and OCT data in combination with machine learning techniques. To solve these problems, it is necessary to divide the method into five steps: data preprocessing, Variable selection, model building, cross-validation and model assessment (see Fig. 2).

#### Data Preprocessing

Data preprocessing is a very important step in machine learning. Data cleaning, missing data resolution and data balancing are included here. We had to eliminate those subjects with incomplete data and remove from the study those variables that had not been collected in a large number of subjects.

Given a binary classification problem, the data are class-imbalanced when the majority of the subjects represent one class. In this way, many classification algorithms have low predictive accuracy for the infrequent class. The problem of class imbalance is closely related to cost-sensitive learning, in which the costs of errors, per class, are not equal. It is much worse to falsely diagnose a MS patient as healthy control (false negative) than to misdiagnose a healthy control as MS patient (false positive). A false negative could result in the loss of life, so is much more expensive than a false positive.

In order to improve the classification performance of class-imbalanced data, synthetic minority over-sampling technique (SMOTE) was used. SMOTE is widely used to balanced clinical data in machine learning approaches.^60^ This method works by resampling the minority class so that the resulting dataset contains an equal number of positive and negative subjects. To increase the sample of the minority class, SMOTE synthesises new cases. To do so, a data point is randomly selected from the minority class and its k-nearest neighbours (k-NN) are determined. Following the consensus, 5 neighbours were used. The new synthetic subject is a combination of the randomly selected data point and its neighbours.^24^

#### Variable Selection

In the development of predictive models, the selection of relevant variables has several advantages such as reducing overfitting, improving predictive accuracy and reducing computational cost. In machine learning, a rule of thumb is to have a number of subjects per class of at least ten time the number of variables.^39^ To perform this variable selection, least absolute shrinkage and selection operator (LASSO) and sequential forward selection (SFS) were used to remove the irrelevant variables. LASSO regression imposes a constraint on the model variables that produces regression coefficients so that some of these variables are reduced to zero and removed from the dataset, retaining only the good features of the data.^57^ SFS method is based on trying to minimize the objective function called misclassification rate over all possible subsets of features. To minimize this rate, this sequential forward algorithm incorporates features while evaluating the objective function until adding more features does not decrease the objective function.^54^

#### Model Building

The predictive models were developed for two purposes: MS diagnosis and MS prognosis. The classifiers used for model building were implemented in Matlab (version 2020b, Mathworks Inc., Natick, MA) using the Statistics and Machine Learning Toolbox. Classifier performance was optimised by hyperparameter optimization, which attempts to minimise the cross-validation loss.

##### Multiple Linear Regression

Multiple linear regression (MLR) is used to estimate the relationship between one or more explanatory variables and a response variable by fitting a linear equation to observed data. MLR can be very useful in understanding the role that predictors play in the predictive model.^38^ This is the simplest linear model to be tested in a binary classification problem.^32^

##### Support Vector Machine

In a binary classification problem, SVM seeks the optimal hyperplane that separates two different classes with the maximum margins. SVM supports the mapping of predictor data using kernel functions with an optimised kernel scale value to increase the separability of the hyperplane for non-separable problems.^58^ In case of non-separable classes, this classifier imposes a penalty factor, called box constraint, whose aim is to avoid overfitting. This classifier has been extensively tested in the literature, both for MS diagnosis^9,41^ and MS prognosis.^65^

##### K-Nearest Neighbours

The k-NN algorithm is one of the most used classifiers in machine learning.^10,21,33^ This algorithm consists in associating the training data with a distance function and the class choice function based on the classes of nearest neighbours. Before classifying a new subject, it should be compared with another subject using a similarity measure. Its k-nearest neighbours are considered and the class that appears most among them is assigned to the new subject. In general, a number of neighbours greater than one is used, since such a small number could lead to overfitting. The neighbours are weighted by the distance from the new subjects to be classified.^63^

##### Decision Tree

In the DT classifier, a tree is developed and it contains a predefined target variable. The structure of a DT contains a root node, several internal nodes and several leaf nodes. This tree is traversed from root to leaf for decision making and this process is carried out until the criteria are met.^10^ The minimum number of leaf node observations and the minimum number of branch observations are the parameters with which the depth of the trees can be controlled. The ability of DT to accurately classify between MS patients and healthy controls and to predict the short-term course of MS has also been previously investigated.^3,63^

##### Naïve Bayes

Based on Bayesian theory for density estimation, the Naïve Bayes (NB) classifier assumes that predictor variables are independent of each other. This assumption of independence increases the simplicity of the model. Kernel density estimation, defined by the smoothing parameter called bandwidth, is one of the most commonly used data distributions. The choice of this hyperparameter determines the smoothness of the density plot, so it is preferable to choose a bandwidth as small as the data allow. The performance of the NB algorithm is comparable to that of the DT due to its high accuracy and speed, as well as fast training and low computational complexity.^20^

##### Ensemble Classifier

Another possibility is to combine several algorithms using ensemble methods. EC generates several base classifiers from which a new classifier is derived which works better than any constituent classifier. The motivation is to combine weak models to produce a powerful ensemble.^24^ LogitBoost was used as the ensemble aggregation algorithm to train the set of boosted classification trees. There are several hyperparameters to optimize the performance of this classifier: number of learning cycles, learning rate and minimum number of leaf node observations.^5^ In this structure, the number of learning cycles corresponds to the number of classification trees. The learning rate limits the contribution of each new classification tree added in the algorithm. These type of ensemble learning approaches showed good performance in previous studies with the same purposes as this work.^36,65^

##### Long Short-Term Memory

Recurrent neural network (RNN), particularly those that work by learning sequences, such as long short-term memory (LSTM), are very useful in the context of disability course prediction. In previous works comparing several classifiers with this objective, this method showed the best results.^36,52^ LSTM models are able to work with long-range dependencies and non-linear dynamics. Another sequence models, such as Markov models, conditional random fields and Kalman filters, deal with sequential data but fail to learn the long-range dependencies. However, this RNN can learn representations and can discover unexpected structures.^28^ The LSTM neural network implemented in this work had the following structure: a sequence input layer, a bidirectional LSTM layer with predefined hidden layers containing the information recalled between time steps, a fully connected layer, a softmax layer and a final classification output layer. The input layer inputs the features into the network. The size of the fully connected layer correspond to the number of classes. Finally, softmax layer converts a vector of real values into a vector of probabilities. This structure can be improved by optimising the number of hidden layers, the epochs and the mini-batch size. A mini-batch is a subset of the training set used to evaluate the gradient of the loss function and update the weights. An epoch is the complete passage of the algorithm over the entire training set using mini-batches.

#### Cross-Validation

Since our dataset was not large enough to use hold-out validation, k-fold cross-validation was used to reduce the risk of overfitting. In addition, this method ensures that the result is independent of the initial division.^46^ The data set was randomly divided into equal k-fold, using 1-fold as a test set and the remaining folds as training set. This process is repeated k-times until each fold has been used as a test set and the overall performance is calculated by the combination of these k-iterations. A 10-fold cross-validation was used, as it is the general recommendation in the machine learning field due to its balance between performance and computational cost.^20^

Data normalization was performed to improve the quality of our dataset. The normalization used for numerical variables consists of the normalization of the training set (mean of 0 and standard deviation of 1) and the normalization of the test set using the mean and standard deviation of the training set. With this method, the classification algorithms do not have access to future information. Since these algorithms work with numerical variables, categorical variables, such as sex, MS subtype, optic neuritis antecedent and relapse in preceding year, had to be encoded into numerical values using one-hot encoding.^44^

### Model Assessment

Confusion matrix was used as a performance measurement because it is extremely useful to determine accuracy, sensitivity, specificity, precision and negative predictive value (NPV). First, four parameters have to be defined: true positives (TPs) are the positive data correctly classified and true negatives (TNs) are the negative data correctly classified, false positives (FPs) are the negatives classified as positives and false negatives (FNs) are the positives classified as negatives. Accuracy provides the percentage of correctly classified subjects. Sensitivity is used to determine the proportion of positives that are correctly identified and specificity is used to determine the proportion of negatives that are correctly identified. Precision expresses the percentage of the predicted positives that are actually positive and NPV is the percentage of the predicted negatives that are actually negatives.

1 $$\mathrm{Accuracy }(\mathrm{acc})=\frac{\mathrm{TP}+\mathrm{TN}}{\mathrm{TP}+\mathrm{TN}+\mathrm{FP}+\mathrm{FN}},$$

2 $$\mathrm{Sensitivity }\left(\mathrm{sens}\right)=\frac{\mathrm{TP}}{\mathrm{TP}+\mathrm{FN}},$$

3 $$\mathrm{Specificity} \left(\mathrm{spec}\right)=\frac{\mathrm{TN}}{\mathrm{TN+FP}},$$

4 $$\mathrm{Precision} \left(\mathrm{prec}\right)=\frac{\mathrm{TP}}{\mathrm{TP}+\mathrm{FP}},$$

5 $$\mathrm{Negative predictive value} (\mathrm{NPV})=\frac{\mathrm{TN}}{\mathrm{TN}+\mathrm{FN}},$$

There are more parameters to evaluate a binary classification. F1 score is the harmonic mean of precision and sensitivity and Fowkles–Mallows index (FM) is the geometric mean of precision and sensitivity. However, these parameters do not take into account TN and give equal importance to precision and sensitivity when, in practice, different misclassifications cause different costs. For example, a FN (MS patient classified as healthy control) is worse than a FP (healthy control classified as MS patient). To solve that, we used Matthews correlation coefficient (MCC), which is a correlation coefficient between actual values and predicted values. It ranges from -1 to 1, where 0 indicates a random classification. Since there is no perfect way to describe the confusion matrix by a single number, this parameter is one of the most informative because it takes into account true and false positives and true and false negatives.

6 $$F1\,\mathrm{score}=2\frac{\mathrm{Prec}*\mathrm{Sens}}{\mathrm{Prec}+\mathrm{Sens}},$$

7 $$\mathrm{FM}=\sqrt{\mathrm{Prec}*\mathrm{Sens}},$$

8 $$\mathrm{MCC}=\frac{\mathrm{TP}*\mathrm{TN}-\mathrm{FP}*\mathrm{FN}}{\sqrt{(\mathrm{TP}+\mathrm{FP})(\mathrm{TP}+\mathrm{FN})(\mathrm{TN}+\mathrm{FP})(\mathrm{TN}+\mathrm{FN})}},$$

Another interesting parameter is Cohen’s kappa coefficient (*κ*), which is used to determine the degree of agreement between actual and predicted values. It is a more robust measure because it takes into account the possibility of a correct classification by chance.

9 $$\kappa =\frac{\mathrm{Acc}-\mathrm{Random}\,\mathrm{acc}}{1-\mathrm{Random\,acc}},$$

10 $$\mathrm{Random\,Acc}=\frac{\left(\mathrm{TN}+\mathrm{FP}\right)\left(\mathrm{TN}+\mathrm{FN}\right)+(\mathrm{FN}+\mathrm{TP})(\mathrm{FP}+\mathrm{TP})}{{(\mathrm{TP}+\mathrm{TN}+\mathrm{FP}+\mathrm{FN})}^{2}},$$

The receiver operating characteristic (ROC) curve, a graph that illustrates the diagnostic ability of a classification algorithm, was also analysed. The ROC curve is drawn by plotting the true positive rate (TPR) or sensitivity against the false positive rate (FPR) as the discrimination threshold is varied. The area under the curve (AUC) provides a measurement of performance at all possible classification threshold.

### MS Diagnosis Model

A MS diagnosis model was developed using the data from 72 MS patients and 30 healthy controls evaluated in our cross-sectional study. For this model, we used three datasets, one per each protocol. **Dataset 1**: general data and fast macular thickness protocol (13 features). **Dataset 2**: general data and fast RNFL thickness protocol (778 features). **Dataset 3**: general data and fast RNFL-N thickness protocol (780 features).

**Figure 3:**
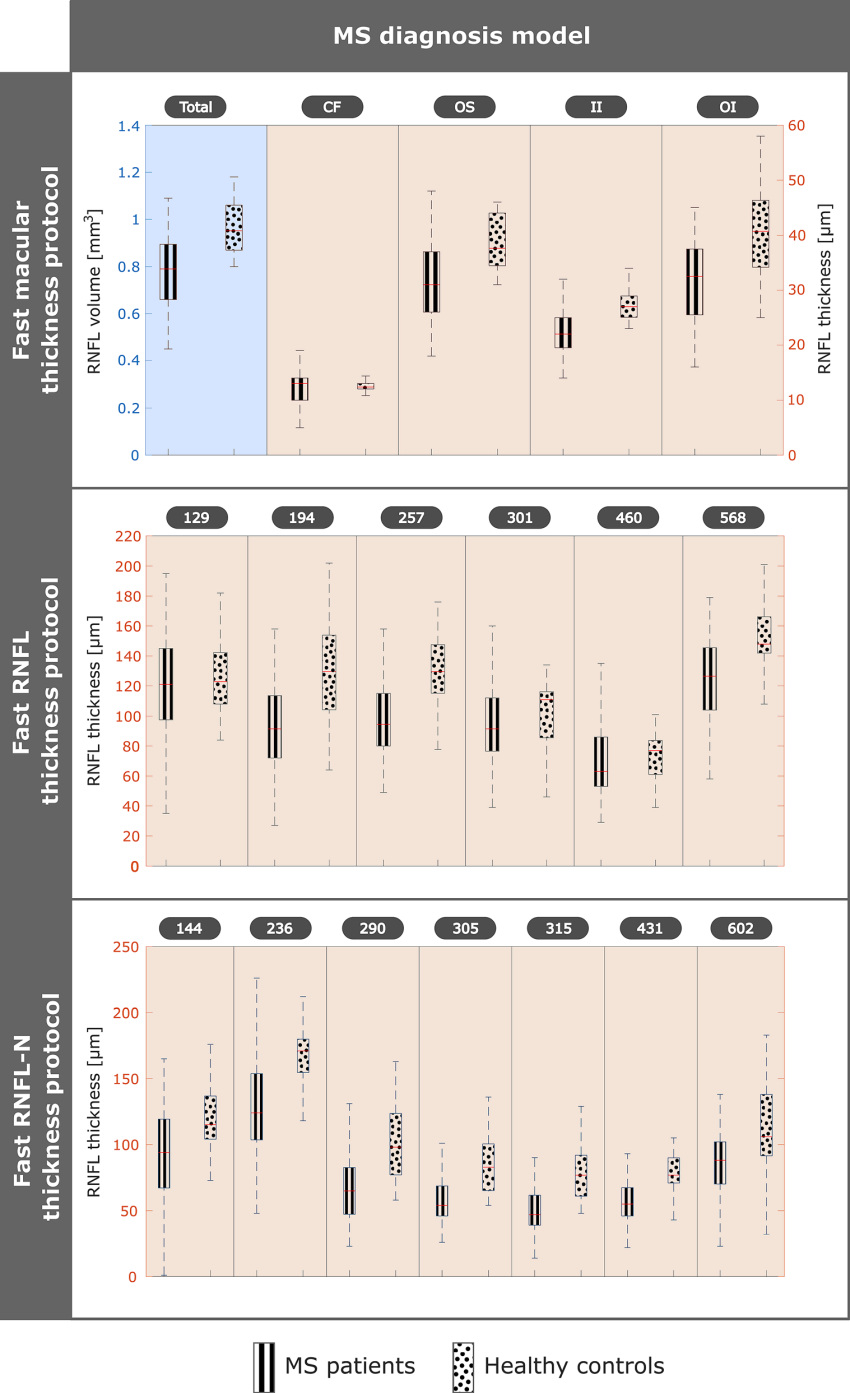
Variable selection for multiple sclerosis (MS) diagnosis model after applying least absolute shrinkage and selection operator (LASSO) to balanced data with 72 MS patients and 72 healthy controls. Raw dataset 1 included general data and fast macular thickness protocol (13 features), raw dataset 2 included general data and fast retinal nerve fiber layer (RNFL) thickness protocol (778 features), and raw dataset 3 included general data and fast RNFL-N thickness protocol (780 features). *CF* central fovea, *OS* outer superior, *II* inner inferior, *OI* outer inferior.

This cross-sectional study was class-imbalanced, so SMOTE was used to resample healthy controls class. In this way, the data turned out to be 72 MS patients and 72 healthy control, a total of 144 subjects. It can be seen that these three datasets contained too many variables compared to the number of subjects per class, so it was necessary to perform variable selection. LASSO was applied to reduce the datasets to five, six or seven variables, depending on the dataset (see Fig. 3). As previous works also demonstrated,^29,36,37^ the reduced datasets after applying LASSO showed a better model performance. Finally, the six classifiers were tested using the 10-fold cross-validation for model assessment. The LSTM was not used in this model because it is designed to work with time series.

### MS Prognosis Model

Here, the data from our longitudinal study were used to develop a model capable or predicting the long-term course of disability state in MS patients. The 72 MS patients were evaluated in seven visits: a baseline visit followed by five annual visits and a final visit 10 year after the start of the follow-up. This model was carried to know the disability state of MS patients in the future, distinguishing between patients whose disability state will get worse and patients whose disability state will remain in a similar neurological state. We established, following the standard definition of disability progression,^22^ that a MS patient gets worse when the criteria shown in Table 2 are met between the target future time and the time the prediction is made. In contrast, MS patients whose EDSS values do not meet the standard criteria are considered patients who remain in a similar disability state. We proposed to make a prediction as soon as possible, for this reason, we developed a first model using the data from the first 2 years of the follow-up to predict the disability state 9 years later. These two data points are the minimum necessary for the classifiers to have a sequence to work with. We developed a second model using data from the first 3 years to evaluate whether delaying the prediction by 1 year leads to an increase in the model performance. With this second model, the disability state is predicted 8 years later.

**Table 2:** Standard criteria for disability progression in multiple sclerosis patients based on expanded disability status scale (EDSS).

Reference EDSS	Criteria
0	An increase of 1.5 or more points in EDSS (∆EDSS ≥ 1.5)
1 to 5.5	An increase of 1 or more points in EDSS (∆EDSS ≥ 1)
6 and up	An increase of 0.5 or more points in EDSS (∆EDSS ≥ 0.5)

Taking into account these considerations, MS patients turned out to be 32 patients with disability progression and 40 patients without disability progression in both models. Therefore, we used six datasets, one per protocol in each model. **Dataset 4**: general data, MS data and fast macular thickness protocol (19 features with 2-year follow-up). **Dataset 5**: general data, MS data and fast RNFL thickness protocol (784 features with 2-year follow-up). **Dataset 6**: general data, MS data and fast RNFL-N thickness protocol (786 features with 2-year follow-up). **Dataset 7**: general data, MS data and fast macular thickness protocol (19 features with 3-year follow-up). **Dataset 8**: general data, MS data and fast RNFL thickness protocol (784 features with 3-year follow-up). **Dataset 9**: general data, MS data and fast RNFL-N thickness protocol (786 features with 3-year follow-up).

**Figure 4:**
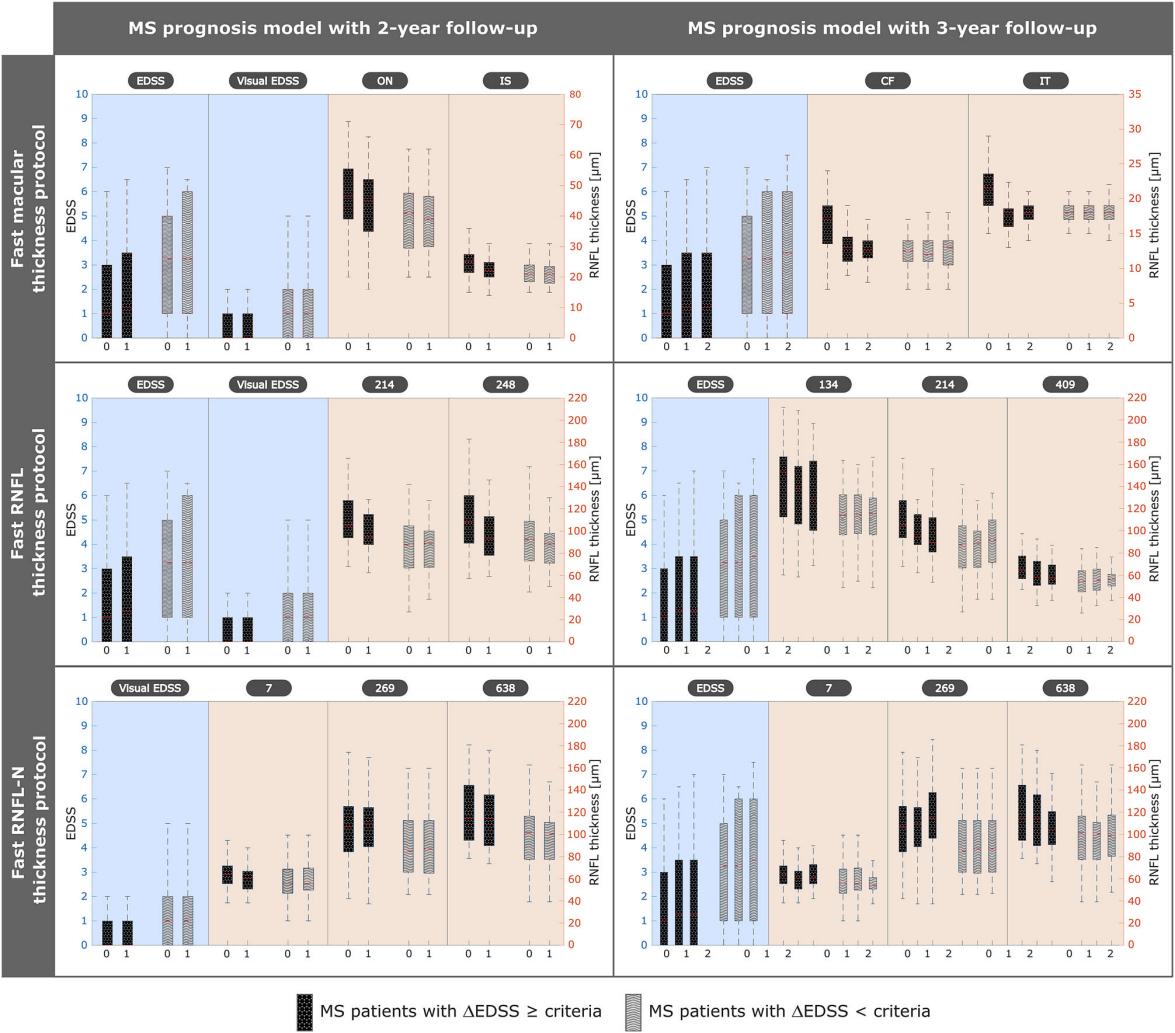
Variable selection for multiple sclerosis (MS) prognosis models after applying least absolute shrinkage and selection operator (LASSO) to balanced data with 40 MS patients with ΔEDSS ≥ criteria and 40 MS patients with ΔEDSS **< ** criteria. Raw dataset 4 included general data, MS data and fast macular thickness protocol (19 features with 2-year follow-up); raw dataset 5 included general data, MS data and fast retinal nerve fiber layer (RNFL) thickness protocol (784 features with 2-year follow-up): and raw dataset 6 included general data, MS data and fast RNFL-N thickness protocol (786 features with 2-year follow-up). Raw dataset 7 included general data, MS data and fast macular thickness protocol (19 features with 2-year follow-up); raw dataset 8 included general data, MS data and fast retinal nerve fiber layer (RNFL) thickness protocol (784 features with 2-year follow-up): and raw dataset 9 included general data, MS data and fast RNFL-N thickness protocol (786 features with 2-year follow-up). Values 0, 1 and 2 on the x-axis represent the years of the 10-year follow-up. *EDSS* expanded disability status scale, *ON* outer nasal, *IS* inner superior, *CF* central fovea, *IT* inner temporal.

As in MS diagnosis model, SMOTE was applied to resample the minority class, in these models, it was the patients with ∆EDSS ≥ criteria. Therefore, the class-balanced data was 40 patients with ∆EDSS ≥ criteria and 40 patients with ∆EDSS < criteria. Another point was the variable selection, in this longitudinal study we had fewer subjects so we had to minimize the number of features, reducing the risk of overfitting and increasing the interpretability. As can be seen in Fig. 4, thanks to LASSO regression, the datasets were reduced to four or even three features.

After the variable selection process, all seven classifiers were evaluated, using 10-fold cross-validation, to determine their capability to predict the long-term course of disability state in MS patients.

## Results

**Figure 5:**
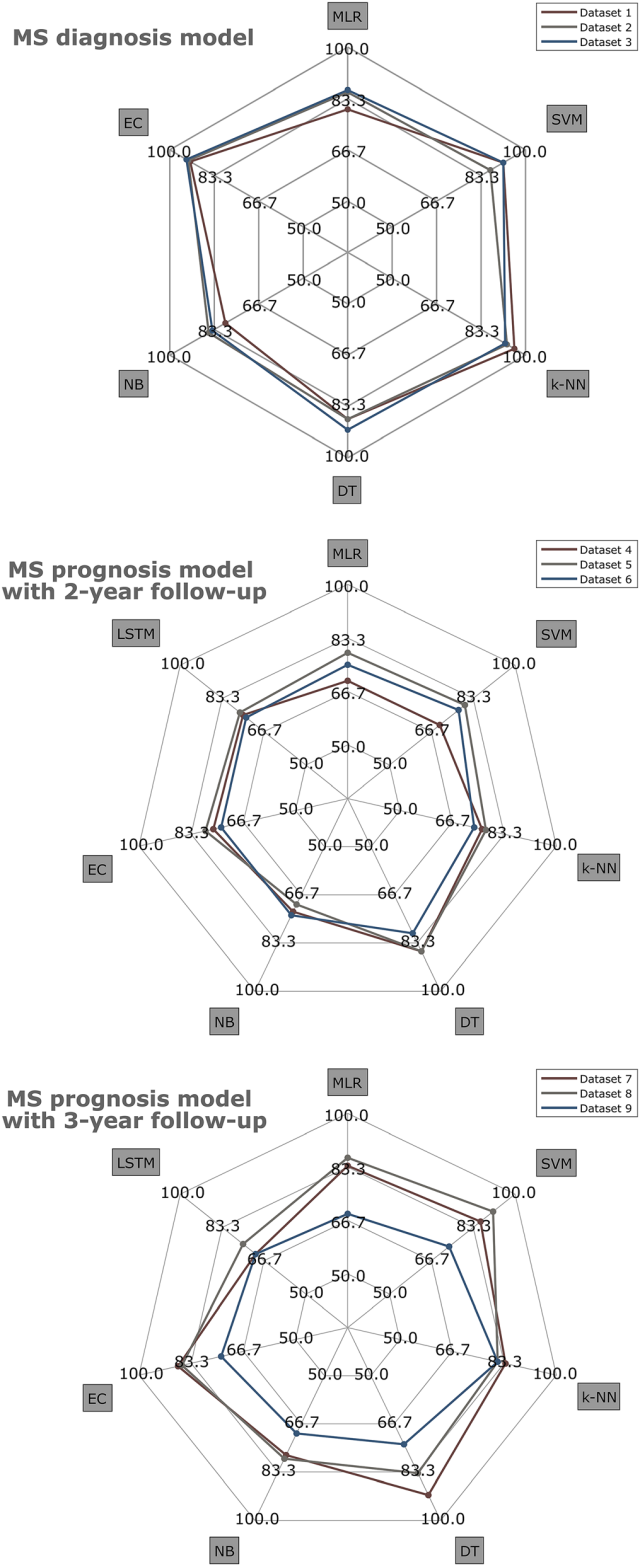
Accuracy of different classifiers for multiple sclerosis (MS) diagnosis and MS prognosis models. Datasets 1, 4 and 7 (brown colour) correspond to clinical data and fast macular thickness protocol. Datasets 2, 5 and 8 (grey colour) correspond to clinical data and fast retinal nerve fiber layer (RNFL) thickness protocol. Datasets 3, 6 and 9 (blue colour) correspond to clinical data and fast RNFL-N thickness protocol. The tested algorithms were: *MLR* multiple linear regression, *SVM* support vector machine, *k-NN* k-nearest neighbours, *DT* decision tree, *NB* Naïve Bayes, *EC* ensemble classifier, *LSTM* long short-term memory, neural network.

Several classifiers were tested to analyse the model performance of these two predictive models using three Spectralis OCT acquisition protocols. The accuracy obtained for all classification algorithms are summarised in Fig. 5.

### MS Diagnosis Model

After balancing the cross-sectional data by SMOTE, variable selection was performed using data from 72 MS patients and 72 healthy controls. As can be seen in Fig. 3, the result obtained with LASSO was as follows: five features (total volume, CF, OS, II and OI) for dataset 1, six features (points 129, 194, 257, 301, 460 and 568) for dataset 2, and seven features (points 144, 236, 290, 305, 315, 431 and 602) for dataset 3. The location of all these features is shown in Fig. 1.

**Figure 6:**
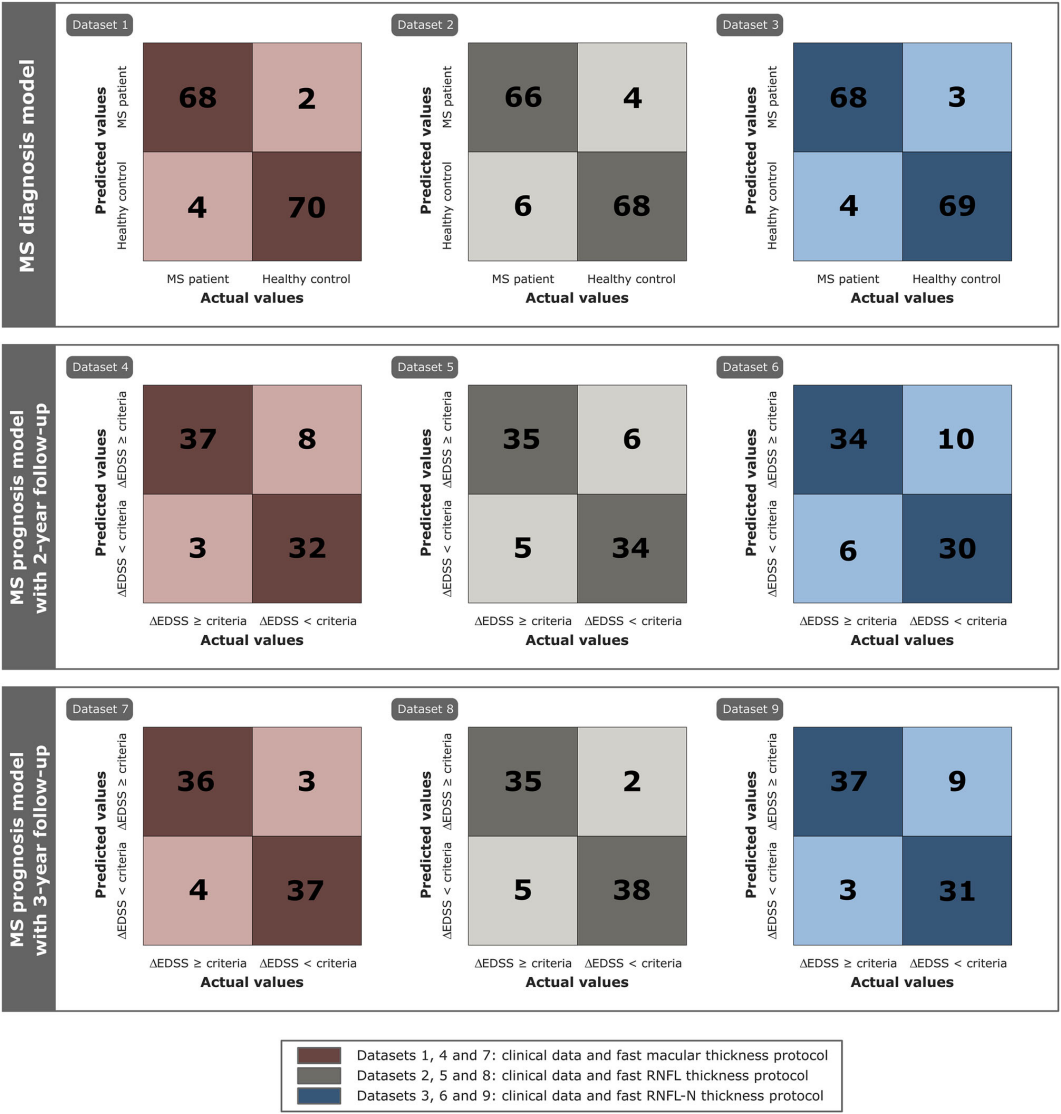
Confusion matrix of the best classifier for each predictive model using different datasets. Top: results for multiple sclerosis (MS) diagnosis. Middle: results for MS prognosis with 2-year follow-up. Bottom: results for MS prognosis with 3-year follow-up. The best classifier and several parameters to analyse the model performance for each dataset were shown in Table 4. (*ΔEDSS* expanded disability status scale variation).

**Figure 7:**
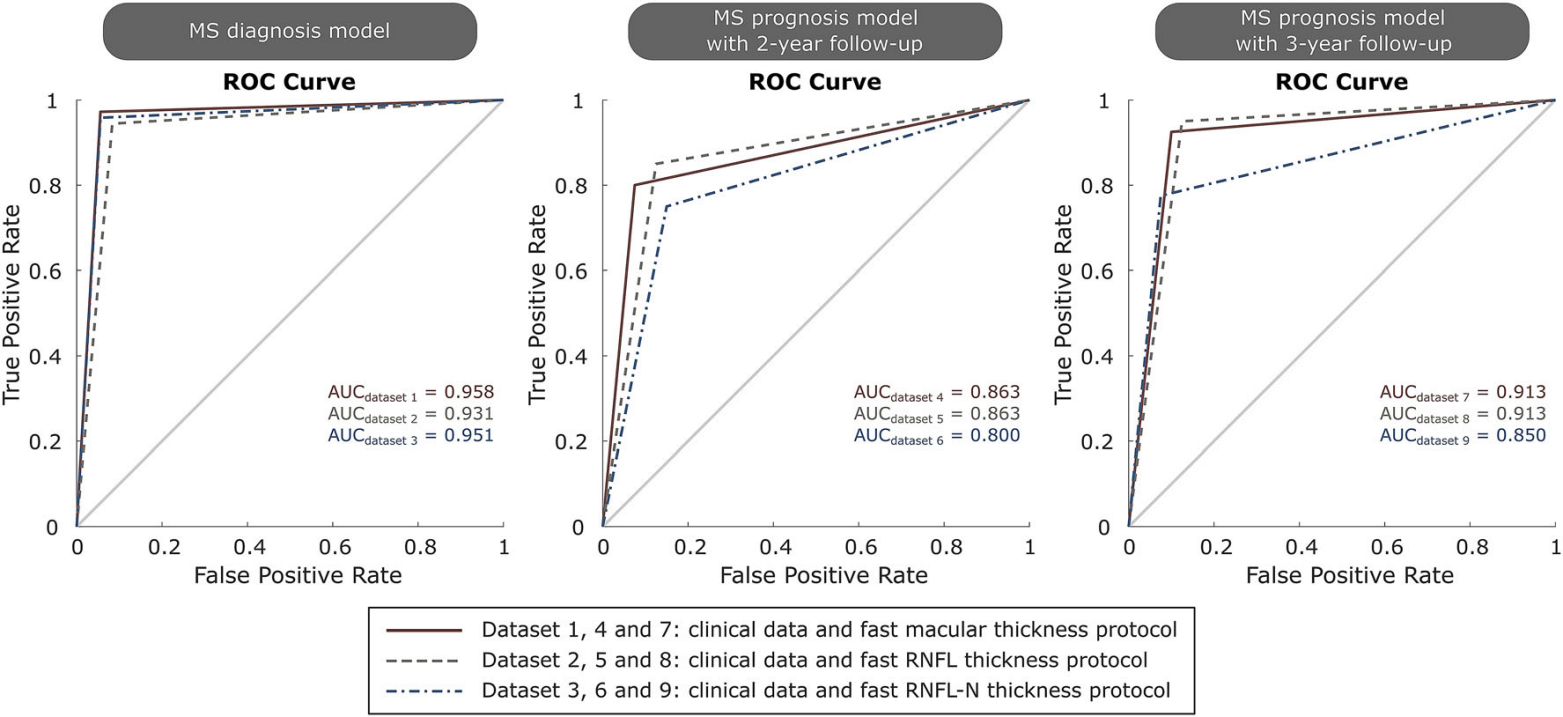
Receiver operating characteristic (ROC) curve with area under curve (AUC) of the best classification algorithm for multiple sclerosis (MS) diagnosis and MS prognosis using different datasets. The best classifier and several parameters to analyse the model performance for each dataset were shown in Table 4.

For dataset 1, the best accuracy (95.8%) was obtained using k-NN with 4 as number of nearest neighbours and Euclidean distance as distance metric between neighbours. Looking at Table 1, the variables chosen by LASSO showed a statistically significant difference (*p* < 0.05) between MS patients and healthy controls. In case of dataset 2, k-NN and EC correctly classified 134 out of 144 (4 FPs and 6 FNs, see confusion matrix in Fig. 6), giving an accuracy of 93.1%. The optimal hyperparameters were: 3 nearest neighbours with cosine distance metric for k-NN, and 100 learning cycles, 0.487 learning rate and the minimum of 1 leaf node observation for EC. Finally, for dataset 3, the best classifier was EC with an AUC of 0.951 (see ROC curve in Fig. 7). In this case, its optimal configuration was 65 classification trees, a learning rate of 0.033 and a minimum of 4 observations per leaf node. It can be seen that AUC is equal to accuracy since raw data was balanced in the data preprocessing step.

### MS Prognosis Model

For MS prognosis, two predictive models were proposed: the first used data from the first two years of follow-up to predict disability state 9 years later and the second added one more data point to predict disability progression 8 years later. With this second model, it can be assessed whether delaying the prediction by 1 year increases the model performance. After resampling the minority class, the class-balanced data was 40 MS patients with ΔEDSS ≥ criteria and 40 MS patients with ΔEDSS < criteria.

For clinical data and fast macular thickness protocol, variable selection turned out to be four features (EDSS, visual EDSS, ON and IS) for the first model (dataset 4) and three features (EDSS, CF and IT) for the second model (dataset 7). As can be seen in Fig. 4, EDSS was chosen for both models. The difference between patients with disability progression and without disability progression was significant for EDSS and visual EDSS at the three data points, while for ON, IS, CF and IT was significant only at the baseline (see Table 3). For datasets with fast RNFL thickness protocol, four features (EDSS, visual EDSS, points 214 and 248) were selected in dataset 5 using 2-year follow-up and four features (EDSS, points 134, 214 and 409) in dataset 8 using 3-year follow-up. In these two datasets, both EDSS and point 214 were in the feature selection performed by LASSO. Finally, with data from fast RNFL-N thickness protocol, variable selection was almost the same for both models: four features (visual EDSS, points 7, 269 and 638) to predict disability progression 9 years later (dataset 6) and four features (EDSS, points 7, 269 and 638) to predict 8 years later (dataset 9).

**Table 3:** General data, multiple sclerosis (MS) data and retinal nerve fiber layer (RNFL) data, measured by Spectralis optical coherence tomography (OCT), from 32 MS patients with ∆EDSS ≥ 1 and 40 MS patients with ∆EDSS < 1 at the first three years of the 10-year follow-up.

	MS patients ∆EDSS ≥ crit. (*n *= 32)	MS patients ∆EDSS < crit. (*n *= 40)	*p*-value	MS patients ∆EDSS ≥ crit. (*n *= 32)	MS patients ∆EDSS < crit. (*n *= 40)	*p*-value	MS patients ∆EDSS ≥ crit. (*n *= 32)	MS patients ∆EDSS < crit. (*n *= 40)	*p*-value
	Baseline			1 year			2 years		
General data									
Age (years)	44.01 ± 10.30	44.77 ± 12.51	0.905	45.11 ± 9.19	45.93 ± 12.21	0.9413	46.38 ± 9.76	47.14 ± 11.74	0.887
Sex (M–F)	12–20	7–33		12–20	7–33		12–20	7–33	
BCVA (Snellen)	0.93 ± 0.24	0.90 ± 0.27	0.235	0.96 ± 0.12	0.89 ± 0.27	0.073	0.97 ± 0.13	0.92 ± 0.28	0.538
MS data									
MS duration (years)	11.09 ± 5.65	12.19 ± 8.24	0.716	12.37 ± 5.98	13.35 ± 8.05	0.860	13.46 ± 6.23	14.47 ± 8.33	0.977
MS subtype (RRMS–SPMS–PPMS)	29–2–1	36–3–1		29–2–1	35–4–1		29–2–1	35–4–1	
Optic neuritis antecedent (Yes–No)	6–26	9–31		6–26	10–30		7–25	10–30	
Relapse in preceding year (Yes–No)	4–28	6–34		3–29	7–33		1–31	6–34	
EDSS	1.88 ± 1.89	3.45 ± 2.34	**0.004**	1.92 ± 1.87	3.41 ± 2.44	**0.011**	1.97 ± 2.09	3.59 ± 2.45	**0.005**
Visual EDSS	0.63 ± 0.94	1.20 ± 1.16	**0.013**	0.63 ± 0.91	1.15 ± 1.10	**0.014**	0.63 ± 0.87	1.18 ± 1.11	**0.011**
Fast macular thickness protocol									
Total volume (mm^3^)	0.85 ± 0.17	0.72 ± 0.14	**0.002**	0.81 ± 0.17	0.73 ± 0.14	**0.034**	0.82 ± 0.17	0.74 ± 0.13	**0.015**
Central fovea th. (*μ*m)	16.09 ± 3.92	12.73 ± 3.12	**< 0.001**	12.88 ± 2.49	12.30 ± 2.72	0.301	12.41 ± 2.21	12.28 ± 2.82	0.833
Inner nasal th. (*μ*m)	23.31 ± 4.26	20.48 ± 3.88	**0.001**	19.94 ± 3.16	20.10 ± 3.61	0.815	19.78 ± 3.22	19.83 ± 3.07	0.873
Outer nasal th. (*μ*m)	44.75 ± 14.02	39.48 ± 10.55	**0.033**	42.91 ± 11.09	39.13 ± 10.34	0.110	43.19 ± 12.45	38.93 ± 9.45	0.089
Inner superior th. (*μ*m)	24.97 ± 4.84	21.35 ± 3.70	**0.001**	21.97 ± 3.90	21.28 ± 3.66	0.403	21.28 ± 3.79	20.98 ± 4.94	0.964
Outer superior th. (*μ*m)	35.88 ± 8.46	26.15 ± 10.79	**< 0.001**	32.22 ± 9.66	26.08 ± 10.66	**0.016**	32.56 ± 7.97	26.55 ± 10.53	**0.029**
Inner temporal th. (*μ*m)	21.50 ± 3.77	18.50 ± 2.62	**< 0.001**	17.44 ± 4.04	18.33 ± 1.91	0.341	17.78 ± 1.86	18.13 ± 2.02	0.474
Outer temporal th. (*μ*m)	22.41 ± 3.49	18.73 ± 2.03	**< 0.001**	18.63 ± 3.92	18.58 ± 1.58	0.216	18.09 ± 3.74	18.73 ± 1.69	0.959
Inner inferior th. [*μ*m)	26.59 ± 4.54	22.65 ± 3.86	**< 0.001**	23.16 ± 4.06	22.28 ± 3.71	0.465	23.13 ± 4.55	22.10 ± 4.04	0.244
Outer inferior th. (*μ*m)	36.81 ± 10.78	27.18 ± 12.49	**0.001**	33.59 ± 9.86	27.50 ± 11.54	**0.019**	34.22 ± 10.50	28.95 ± 9.35	**0.015**
Fast RNFL thickness protocol									
Mean th. (*μ*m)	96.19 ± 22.67	82.85 ± 16.36	**0.002**	91.41 ± 22.50	81.58 ± 15.78	**0.034**	89.31 ± 23.31	81.15 ± 16.13	0.130
Temporal th. (*μ*m)	64.78 ± 20.42	55.23 ± 14.95	**0.039**	59.53 ± 20.03	54.13 ± 14.56	0.310	58.47 ± 18.37	52.13 ± 12.23	0.156
Superotemporal th. (*μ*m)	133.06 ± 41.01	107.13 ± 27.04	**0.002**	127.34 ± 41.06	106.18 ± 25.52	**0.009**	125.59 ± 40.34	104.73 ± 24.81	**0.007**
Inferotemporal th. (*μ*m)	128.53 ± 42.54	112.90 ± 26.86	0.053	124.00 ± 42.69	110.03 ± 28.16	0.132	128.19 ± 43.93	112.83 ± 27.10	0.115
Nasal th. (*μ*m)	77.84 ± 22.37	68.25 ± 23.23	**0.033**	73.75 ± 21.30	66.88 ± 22.44	0.145	69.59 ± 21.68	67.53 ± 22.46	0.555
Superonasal th. (*μ*m)	110.69 ± 23.85	91.70 ± 20.92	**0.002**	103.19 ± 22.98	91.10 ± 20.11	**0.041**	99.34 ± 24.31	92.25 ± 22.61	0.292
Inferonasal th. (*μ*m)	112.19 ± 34.56	103.10 ± 28.32	0.324	109.78 ± 34.60	102.38 ± 29.39	0.518	105.22 ± 34.52	98.98 ± 30.46	0.830
Fast RNFL-N thickness protocol									
Mean th. (*μ*m)	93.88 ± 20.50	81.89 ± 15.12	**0.009**	90.34 ± 20.04	81.58 ± 14.71	0.093	86.78 ± 24.63	77.82 ± 19.61	0.062
PMB th. [*μ*m)	49.38 ± 15.53	44.84 ± 11.49	0.233	46.13 ± 14.75	44.29 ± 11.21	0.688	44.41 ± 13.40	43.45 ± 11.08	0.813
N/T ratio	1.36 ± 0.53	1.27 ± 0.40	0.560	1.28 ± 0.68	1.19 ± 0.52	0.795	1.32 ± 0.69	1.25 ± 0.48	0.855
Superonasal th. (*μ*m)	110.56 ± 32.16	94.50 ± 21.73	**0.018**	105.88 ± 31.77	93.47 ± 23.51	0.139	99.81 ± 32.64	88.92 ± 28.34	0.273
Nasal th. (*μ*m)	77.97 ± 24.77	65.37 ± 15.42	**0.024**	74.06 ± 24.56	66.18 ± 14.31	0.209	70.28 ± 26.27	63.58 ± 18.44	0.342
Inferonasal th. (*μ*m)	108.75 ± 33.08	104.92 ± 26.86	0.958	105.00 ± 34.06	103.74 ± 25.51	0.773	103.06 ± 27.77	97.11 ± 31.90	0.571
Inferotemporal th. (μm)	126.09 ± 28.19	108.63 ± 28.80	**0.007**	123.97 ± 27.31	109.32 ± 29.81	**0.020**	126.63 ± 28.07	109.00 ± 33.24	**0.027**
Temporal th. (*μ*m)	60.97 ± 16.49	55.03 ± 15.17	0.122	58.03 ± 15.78	53.47 ± 14.35	0.186	57.66 ± 16.94	52.42 ± 13.44	0.144
Superotemporal th. (*μ*m)	127.50 ± 30.17	106.21 ± 26.83	**0.003**	123.81 ± 30.07	106.74 ± 26.29	**0.012**	123.94 ± 30.56	107.13 ± 26.29	**0.019**

∆EDSS represents the variation of expanded disability status scale (EDSS) between the target future time and the time the prediction is made. The criteria for disability progression are show in Table 2. P-value, obtained by Wilcoxon test, is used to compare data between MS patients with ∆EDSS ≥ criteria and MS patients with ∆EDSS < criteria. Statistically significant differences (*p* < 0.05) are represented in bold.

*BCVA* best-corrected visual acuity, *RRMS* relapsing-remitting multiple sclerosis, *SPMS* secondary-progressive multiple sclerosis, *PPMS* primary-progressive multiple sclerosis, *th* thickness, *PMB* papillomacular bundle, *N/T* nasal/temporal

First, we evaluated the ability of these classifiers to predict whether or not a MS patient will get worse using data from three Spectralis OCT acquisition protocols collected at the first 2  years (see Fig. 5 for accuracy of all classifiers). The best result was an 86.3% accuracy obtained by DT in dataset 4 (minimum of 1 observation per leaf node and 10 observations per branch) and in dataset 5 (minimum of 4 observations per leaf node and 10 observations per branch). As can be seen in the confusion matrices, there were 3 FNs in dataset 4 compared to 5 FNs in dataset 5. For dataset 6, the best classifier was also DT with an accuracy of 80.0% and its hyperparameters were a minimum of 6 leaf node observations and 10 branch observations.

Second, adding an additional data point to the previous model, we tested whether delaying the prediction by 1 year results in an increase in the model performance. For dataset 7, the predictions generated by DT were correct in 73 of 80 cases (3 FPs and 4 FNs, see confusion matrix in Figure 6) giving an accuracy of 91.3%. The same accuracy and AUC were obtained for dataset 8 using SVM whose optimal structure was a box constraint of 0.431 and a kernel scale of 0.109. Finally, k-NN correctly classified 68 out of 80 MS patients using dataset 9 with an AUC of 0.850 (see Fig. 7). The hyperparameter optimization showed 4 neighbours as the optimal number of nearest neighbours and Euclidean distance as the distance metric between them.

## Discussion

In MS, many factors influence the development and progression of this disease so that even large correlational studies have come to weak conclusions.^48^ Therefore, it is time to take advantage of the potential of data-driven ML analysis. Most ML approaches were based on the MRI examination to diagnose MS or to predict disease progression, following the emerging use of image analysis.^1^ However, we propose a ML approach to diagnose MS and provide long-term predictions of disability progression based on RNFL thickness measured by OCT. This imaging technique has some advantages over MRI since it is a fast, cost-effective and non-invasive test.

Paying attention to the statistical analysis of raw data between MS patients and healthy controls (see Table 1), the difference was significant in almost all features (not for CF and IT) for fast macular thickness protocol, in all features for fast RNFL thickness protocol and in most features (not for mean thickness and N/T ratio) for fast RNFL-N thickness protocol. In relation to general data, the difference was also significant in BCVA. As previous studies have shown,^4,15^ axonal loss affects the entire pRNFL, with the temporal quadrant being the most affected area in MS patients. It can also be observed that mRNFL showed a significant decrease in this disease. Fig. 3 shows the variables selected by LASSO to develop the MS diagnosis model after balancing our raw data. As expected, the general trend was that both volume and thickness were higher in healthy controls than in MS patients. It is well known that RNFL thinning occurs as part of normal aging,^59^ but an additional thinning occurs as a pathological consequence of MS. In the early stages of the disease, demyelination and axonal transection occur. And, as the pathology progresses, inflammation and axonal degeneration predominate.^25,35^

In our MS diagnosis model, the best accuracy was obtained with fast macular thickness protocol (database 1), very similar to that obtained with fast RNFL-N thickness protocol (database 3). And the best classifiers for this purpose were k-NN and EC (see Table 4). This result (acc: 95.8%; AUC: 0.958) was better than that obtained in previous works by Garcia-Martin *et al*. who also used Spectralis OCT: an AUC of 0.945^18^ and an accuracy of 88.5%^16^ using ANN. Compared to studies that used SS-OCT Triton to measure RNFL, Pérez del Palomar *et al*.^41^ obtained an accuracy of 97.2% using DT, Cavaliere *et al*.^9^ 90.6% using SVM and Garcia-Martin *et al*. ^17^ 97.9 using ANN. In our previous work^36^, the best result was an accuracy of 87.7% using also EC with Cirrus HD-OCT data.

**Table 4:** Model assessment for each dataset, only the best classifier is shown.

Dataset	Features	Classifier	Acc (%)	Sens (%)	Spec (%)	Prec (%)	NPV (%)	F1 score	FM	MCC	*κ*	AUC
MS diagnosis model												
1	5	k-NN	95.8	94.4	97.2	97.1	94.6	0.958	0.958	0.917	0.917	0.958
2	6	k-NN/EC	93.1	91.7	94.4	94.3	91.9	0.930	0.930	0.861	0.861	0.931
3	7	EC	95.1	94.4	95.8	95.8	94.5	0.951	0.951	0.903	0.903	0.951
MS prognosis model with 2-year follow-up												
4	4	DT	86.3	92.5	80.0	82.2	91.4	0.871	0.872	0.731	0.725	0.863
5	4	DT	86.3	87.5	85.0	85.4	87.2	0.864	0.864	0.725	0.725	0.863
6	4	DT	80.0	85.0	75.0	77.3	83.3	0.810	0.810	0.603	0.600	0.800
MS prognosis model with 3-year follow-up												
7	3	DT	91.3	90.0	92.5	92.3	90.2	0.911	0.911	0.825	0.825	0.913
8	4	SVM	91.3	87.5	95.0	94.6	88.4	0.909	0.910	0.827	0.825	0.913
9	4	k-NN	85.0	92.5	77.5	80.4	91.2	0.860	0.863	0.708	0.700	0.850

As can be seen in Eqs. [1–9], the following parameters are calculated: accuracy (acc), sensitivity (sens), specificity (spec), precision (prec), negative predictive value (NPV), F1 score, Fowkles-Mallows index (FM), Matthews correlation coefficient (MCC), Cohen’s Kappa coefficient (*κ*). Area under curve (AUC) is the area under the receiver operating characteristic (ROC) curve

*k-NN* k-nearest neighbours, *EC* ensemble classifier, *DT* decision tree, *SVM* support vector machine

For MS prognosis, MS patients of our longitudinal study were divided into two classes based on standard criteria for disability progression (Table 2). Table 3 shows the statistical analysis of clinical data and RNFL data performed between 32 MS patients with disability progression and 40 MS patients without disability progression at the first 3 year of our follow-up (the first two for the first model and the first three for the second model). For MS data, the difference was significant in EDSS and visual EDSS at the 3 years. In fast macular thickness protocol, the difference turned to be significant in all features at baseline, while only in total volume, OS and OI at visits 1 and 2. In fast RNFL thickness protocols, the difference between classes was found to be significant in mean thickness, T, ST, N, SN at baseline; in mean thickness, ST and SN at year 1; and in ST at year 2. Finally, for fast RNFL-N thickness protocol, the difference was significant in mean thickness, SN, N, IT and ST at baseline, and in IT and ST at years 1 and 2. With these results, it could be said that the difference in RNFL thickness was higher at the baseline visit of our 10-year follow-up.

After applying LASSO regression to the class-balanced data (40 MS patients with ΔEDSS ≥ criteria and 40 MS patients with ΔEDSS < criteria), the variable selection was shown in Fig. 4. On the one hand, EDSS and visual EDSS were chosen and it can be seen how MS patients with ΔEDSS < criteria had higher values while the progression was very similar in both groups. On the other hand, the RNFL data of the three Spectralis OCT protocols chosen showed the same overall behaviour. RNFL thickness was higher in MS patients with ΔEDSS ≥ criteria and these patients experienced a greater RNFL thinning because in MS patients without disability progression RNFL thickness was constant or subtly decreased. In this way, we corroborate the conclusion obtained in one of our previous works,^35^ establishing that axonal damage occurs cumulatively from the onset of MS and that most of the RNFL thinning occurs before the appearance of significant disability. MS patients with disability progression show a RNFL thinning while MS patients without disability progression (patients with greater disability) had this thinning in the past (during the early stages of disability).

In our first MS prognosis model with a 2-year follow-up, the best accuracy was 86.3% and it was obtained with fast macular thickness protocol (dataset 4) and with fast RNFL thickness protocol (dataset 5) using DT in both cases. Using an additional data point to the first model, we developed our second MS prognosis model with a 3-year follow-up. In this second model, the performance increased compared to the first model: an accuracy of 91.3% with fast macular thickness protocol (dataset 7) and DT, and with fast RNFL thickness protocol (dataset 8) and SVM. This result obtained in the prediction of disability progression 8 years later (acc: 91.3%; AUC: 0.913) improved our previous result (acc: 81.7%; AUC: 0.816) obtained by LSTM using Cirrus HD-OCT.^36^

Pending further studies that use OCT data in combination with AI for MS prognosis, we have to compare our results with those of studies that used test such as MRI or EP.^53^ Zhao *et al*.^64^ used MRI data to predict disability progression after 2 years using 3-year follow-up and the best result was an accuracy of 71.0% with SVM. Recently, Zhao *et al*.^65^ also achieved an AUC of 0.83 using MRI data from the first 2 years to predict disease course 3 years later. Using EP, Yperman *et al*.^62^ predicted disability progression after 2 years using 2-year time series with an AUC of 0.75. Pinto *et al*.^42^ developed several models to predict disease severity in the 6/10th year of progression using 1-5 years of follow-up with MRI, EP and cerebrospinal fluid (CSF) data. It is clear that model performance increased over time, but it is preferable to achieve a good accuracy with the minimum number of data points. Therefore, these authors considered that the 2-year model (AUC: 0.89) was the most suitable to predict disease severity 4 years later.

As can be seen in Table 4, the results of this work could indicate some conclusions. For MS diagnosis, the best acquisition protocols of Spectralis OCT were fast macular thickness and fast RNFL-N thickness. In addition, the best performing binary classifiers for this task were k-NN and EC, while simpler methods such as MLR or NB showed a performance not as good as the previous ones (see accuracy for MS diagnosis model in Fig. 5). Our results are totally in accordance with our previous work,^36^ in which the behaviour of the tested algorithms was similar. For both MS prognosis models, the best performance was obtained using datasets with fast macular thickness protocol and fast RNFL thickness protocol in combination with DT or SVM. For prognosis purposes, the accuracy increased from 86.3% to 91.3% using one more data point. Therefore, it seems worthwhile to delay the prediction by 1 year to increase the model performance. In this case, Fig. 5 shows how the behaviour of each classifier strongly depends on the acquisition protocol used and the model developed, and no determining conclusion can be drawn. This fact highlights the need for further machine learning studies using RNFL thickness for MS prognosis. Alternatively, as mentioned above, several studies used data from other tests such as MRI, EP or CSF analysis. Zhao *et al*. compared SVM, LR, RF and EC.^64,65^ Seccia *et al*. tested SVM, RF, k-NN, EC and LSTM.^52^ Pinto *et al*. used MLR, SVM, k-NN and DT.^42^ All of them, together with this work, concluded that SVM is one of the best classifiers to predict MS disease course.

Although our results represent a major step forward in the use of OCT to provide valuable information that could help clinician to treat MS better and faster, this work has several limitations. In our study, only good quality scans were selected, but it is not always possible in clinical practice. The models developed are heavily based on OCT data. However, if these data are combined with other previously studied tests such as MRI, EP or CSF analysis, the model performance could be improved. Although the EDSS score is considered the most useful tool to measure MS disability progression, this scale has low reliability and sensitivity.^34^ Our prediction of progression is based on the variation of EDSS score (ΔEDSS), so the output of our models is a qualitative and not a quantitative prediction.

Another highly limiting aspect is the sample population of our study (72 MS patients and 30 healthy controls) which is too small to establish our results as a gold standard. It can be said that the dataset detailed in this work could be representative of the subjects affected by MS since these data follow the trend of this pathology: 73.6% of MS patients were females and RRMS was the most predominant MS subtype. Moreover, the size of our raw data and characteristics such as age or MS duration were similar to those of previous studies.^36,42^ However, more cross-sectional and longitudinal studies with the same aims and with larger sample population will be required to confirm RNFL thickness as a biomarker for early diagnosis and prediction of the disability progression in MS patients.

We must also take into account our class-imbalanced data and the method used to solve this issue. The use of any method of handling imbalanced datasets actually changes the nature of the dataset, and this fact could imply the generality of the results. However, by generating examples similar to existing minority subjects, SMOTE creates broader and less specific decision boundaries that increase the generalizability of the classifiers, increasing their performance.^23,49^ Thus, the risk of overfitting for the majority class and underfitting for the minority class is reduced.

With this work, we support the idea of several authors to use AI in MS and take advantage of its benefits.^1^ For our particular goal, OCT is an objective, reproducible, cost-effective and non-invasive test that can be performed by any clinician in a couple of minutes, without causing any discomfort to the patient. This study can be considered as a proof of concept on the possibility of diagnosing MS and predicting MS disability progression using a machine learning approach with Spectralis OCT data. This work used data from a hospital with the aim of developing models that are ready to test new patients who are undiagnosed or whose progression is unknown. In addition, disease progression was also analysed by accumulating information based on consecutive years. This would be of great benefit to doctors, who would be able to make an early diagnosis and select more specific treatments according to the predicted disability progression of each MS patient.
